# Impacts of Individual Placement and Support (IPS) program of supported employment on employment and psychosocial well-being among individuals with severe mental illness

**DOI:** 10.1192/j.eurpsy.2023.697

**Published:** 2023-07-19

**Authors:** N. Sipilä, K. Appelqvist-Schmidlechner

**Affiliations:** Equality, Finnish Institute for Health and Welfare, Helsinki, Finland

## Abstract

**Introduction:**

Mental health problems increase the risk for unstable career paths and often lead to early transition to disability pensions. Individual Placement and Support (IPS) is an evidence-based program integrated into psychiatric care helping individuals with serious mental illness to find competitive employment. It comprises personalized job search as well as individual in-work support for both the employee and the employer.

**Objectives:**

The Finnish Individual Placement and Support Evaluation Study (2020-2023) aims at investigating the implementation, feasibility as well as perceived benefits and outcomes of the IPS program implemented for the first time broadly in Finland. The present study focuses on the changes observed among program participants in employment status and psychosocial well-being during the 6- and 12-months follow-ups.

**Methods:**

Both quantitative and qualitative data from different stakeholders have been and will be collected. The data collection will be finished at the end of 2022. The presentation focuses on findings from interviews (n=31) and from questionnaire data collected among program participants at baseline and at 6- and 12-months follow-ups. The total sample will comprise approximately 300 program participants (18−64 years of age) diagnosed with a severe mental illness.

**Results:**

Findings on changes in employment status as well as in psychosocial well-being (self-rated health and work ability, mental distress, positive mental health, self-esteem, satisfaction with life, social inclusion, and perceived social support) will be discussed at the congress. The preliminary findings of the study show that about half of the program participants have succeeded in getting employed at least once during the follow-up. However, becoming employed does not always result in increased psychosocial well-being. Work-related stress, meaningless or unsuitable job, problems at the workplace community or fear of being stigmatized may contribute toward decreased psychosocial well-being.

**Conclusions:**

Meaningful work can play an important role in the process of recovery from mental illness. However, individuals with severe mental health problems need support with the working life related concerns and stress especially at the beginning of employment.

**Disclosure of Interest:**

None Declared

